# Expression of steroidogenesis pathway genes in cumulus cells from women with diminished ovarian reserve after gonadotropin administration: A case-control study

**DOI:** 10.18502/ijrm.v22i9.17474

**Published:** 2024-11-14

**Authors:** Zahra Ahmadnia, Fateme Montazeri, Saeideh Dashti, Mohammad Hasan Sheikhha, Marzieh Lotfi

**Affiliations:** ^1^Department of Genetics, Faculty of Medicine, Shahid Sadoughi University of Medical Sciences, Yazd, Iran.; ^2^Abortion Research Center, Yazd Reproductive Sciences Institute, Shahid Sadoughi University of Medical Sciences, Yazd, Iran.; ^3^Research and Clinical Center for Infertility, Yazd Reproductive Sciences Institute, Shahid Sadoughi University of Medical Sciences, Yazd, Iran.

**Keywords:** Ovarian, Reserve, Steroidogenesis, CYP19A1, PKA, GSK3B, Gonadotropin.

## Abstract

**Background:**

Women with diminished ovarian reserve (DOR) respond differently to gonadotropin medications.

**Objective:**

This study investigates the relationship between effective gene expression in the steroidogenesis pathway and gonadotropin responsiveness in DOR.

**Materials and Methods:**

In this case-control study*,* cumulus cells were obtained from women with DOR after gonadotropin administration (n = 20) and normal ovarian reserve (n = 20). They were divided into the following groups, oocyte number < 3 and oocyte number > 3. After RNA extraction and cDNA synthesis, quantitative polymerase chain reaction was performed to assess the expression levels of cytochrome P450 aromatase (*CYP19A1*), protein kinase A(*PKA*)*,* and glycogen synthase kinase 3 beta (*GSK3B*)genes.

**Results:**

The women with DOR had statistically significant lower expression of *CYP19A1* and *PKA* genes in their cumulus cells compared to control group (p = 0.04, and p < 0.001, respectively). There was also lower expression of the *GSK3B* gene in DOR compared to control group, but it was not significant. Although the expression of the *CYP19A1*, *PKA*, and *GSK3B* genes was lower in women with < 3 oocytes compared to women with more oocytes, this difference was not statistically significant.

**Conclusion:**

In conclusion, DOR may be associated with lower expression of *CYP19A1* and *PKA* genes. Also, considering the decrease in the expression of these genes in people with DOR, the expression of these genes can be used as a tool to predict the treatment.

## 1. Introduction 

Diminished ovarian reserve (DOR) is one of the causes of infertility in women and refers to cases where the antral follicle counts (AFC) < 5 and anti-Mullerian hormone (AMH) level < 1.2 ng/ml (1). The term “ovarian reserve” describes the ovaries capacity for reproduction (2). The hereditary form of DOR is rare and accounts for 4–31% of all cases (3). In 90% of cases, the cause is unknown, and the disease known as idiopathic (3, 4).

Ovarian reserve determines the number and quality of ovarian follicles. To maximize the success rate of infertility treatment, it is essential to evaluate the effective signaling pathways on ovarian reserve (5, 6). Women with DOR, have lower sex hormone levels such as estrogen and inhibin, and higher gonadotropin levels such as follicle-stimulating hormone (FSH) and luteinizing hormone (LH) (7). Gonadotropins play an important role in the ovulation and folliculogenesis processes. An imbalance in the secretion of hormones can disturb folliculogenesis. Estradiol synthesis is influenced by the appropriate interaction of growth hormones, gonadotropins, LH, and FSH (8, 9).

Steroidogenesis is an effective process in the growth of ovarian follicles through the production of estrogen. It starts in theca cells, where cholesterol is metabolized into androgens. Granulosa cells, on the other hand, are responsible for changing androgen into estrogen (10, 11). The main signaling pathways related to steroidogenesis are cAMP/ Protein kinase A (*PKA*) and Wnt/ β -catenin. *PKA* is activated by trophic hormones to control steroid hormone production in steroidogenic cells (12). Glycogen synthase kinase 3 beta (*GSK3B*) plays a key role in the regulation of the Wnt/ β -catenin signaling pathway (13).

LH regulates theca cell steroidogenesis by activating the cAMP signaling pathway through a G protein-coupled receptor. FSH controls the granulosa cell's ability to produce estrogen. FSH binding initiates the cAMP pathway, which in turn causes the synthesis of cytochrome P450 aromatase (*CYP19A1*) and 17-hydroxysteroid dehydrogenase in relation to LH signaling. *CYP19A1* converts thecal androstenedione irreversibly into estrone, and HSD17B1 then changes estrone into the more potent estradiol (14, 15).

One of the crucial elements in the management of assisted reproduction technology is controlled ovarian hyperstimulation. Also, ovarian response varies from person to person; for example, poor responders often have a low in vitro fertilization (IVF) success rate and often need a prolonged ovarian stimulation cycle. Poor IVF outcomes can lead to psychological and financial stress (16). Therefore, it is crucial to find good predictive tools for IVF outcomes, which can identify key genes in the signaling pathways involved in folliculogenesis. Inhibition of follicle-stimulating hormone receptors using siRNA has been reported to influence miR-1261, miR-130a-3p, miR-329-3p, miR-185-5p, miR-144–5p and miR-4463 expressions. Genes that are regulated by the abovementioned miRNAs have been identified using bioinformatic tools, and it has been found that overexpression of miR-4463 plays a key role in poor ovarian response in KGN cells. It has been suggested that miR-4463 is involved in poor response to gonadotrophin injection in women (17).

In the current study, the expression of miR-4463 regulated genes involved in the steroidogenesis pathway, in cumulus cells (CC), following gonadotropin administration in women with DOR and control group were evaluated.

## 2. Materials and Methods

Participants in this case-control study were women aged between 20 and 34 yr who referred to Yazd Reproductive Biology Research Institute, Yazd, Iran from November 2021 to December 2022.

The inclusion criteria in the case group were: AMH < 1.2 ng/ml and AFC < 5 (n = 20), and in control group were AMH > 1.2 ng/ml and AFC < 5 (n = 20) that were candidates for IVF/intracytoplasmic sperm injection cycles because of male problems. Participants with absence of uterine anomaly, endometriosis, history of thyroid disease, chromosomal problems that lead to reduced ovarian reserve, and polycystic ovaries were excluded from this study.

In these women, ovulation stimulation was performed using the antagonist protocol. In such a way that 300 IU of human menopausal gonadotropin (Merional, IBSA, Lugano, Switzerland) is given to patients from the second day of the menstrual cycle to the 6 ${\;}^{\text{th}}$ day. Ultrasound was performed on the 7 ${\;}^{\text{th}}$ day of the patients menstrual cycle. This process was repeated for 5–6 days until follicles with a diameter of 12–15 mm were observed. After observing follicles with the appropriate diameter, 0.25 mg/day of gonadotropin releasing hormone antagonist (Cetrotide; Sereno International S.A., Geneva, Switzerland) was injected. Ovulation stimulation was continued until follicles with a diameter of 16–18 mm were observed. After which ovulation is done.

Then, human chorionic gonadotropin (Pregnyl; Organon, Oss, the Netherlands) was injected. After 36 hr, ovulation was performed and CC were isolated from ovarian follicles by a reproductive biologist.

Patients with low ovarian reserves were divided into 2 groups, depending on the response to ovulation stimulation drugs, the number of oocytes less than 3 and more than 3.

### Total RNA extraction and cDNA synthesis

Total RNA was extracted from CCs. RNA extraction was performed using a TRIZOL reagent. RNA concentration, integrity, and purity were analyzed using a Nanodrop spectrophotometer (DeNovix, USA) and agarose gel electrophoresis. Complementary DNA (cDNA) was synthesized using Pars Toos Company kit, Iran according to the manufacturer's instructions.

### Quantitative reverse transcription polymerase chain reaction (RT-PCR)

SYBR Green ROX qPCR Master Mix kit (Ampliqon, Denmark/Eppendorf) was utilized to conduct real-time PCR experiments. The PCR amplification settings included a denaturation stage at 95 C for 2 min, then 40 cycles of annealing and extension at 59 C for 25 sec. Comparative Ct (Ct) analysis was used to examine these results. The expression of the reference gene 45s rRNA and the genes *CYP19A1*, *PKA*, and *GSK3B* were examined. These tests were carried out in triplicate and at least 3 times independently (Table I).

**Table 1:** The primer sequences (5 '→ 3 ' ) used in qPCR

**Primer name**		**Sequence (5** ' ** → 3** ' **)**	**TM**
*CYP19A1*			
	**Forward**	ACACATCTGGACAGGTTGGA	58
	**Reverse**	ATAGCACTTTCGTCCAAAGGG	59
*PKA*			
	**Forward**	AGTACCTGGCCCCTGAGATT	60
	**Reverse**	AGATCTGGATGGGCTGGTCT	60
*GSK3B*			
	**Forward**	ATCTGCCATCGGGATATTAAAC	58
	**Reverse**	ATACGAAACATTGGGTTCTCCT	58
*45 SrRNA*			
	**Forward**	AGAAACGGCTACCACATCCA	59
	**Reverse**	CCCTCCAATGGATCCTCGTT	59
qPCR: Quantitative polymerase chain reaction,* CYP19A1*:Cytochrome P450 aromatase 19 subfamily A member 1,* PKA*: Protein kinase A, *GSK3B*: Glycogen synthase kinase 3 beta, *45 SrRNA*: 45s ribosomal RNA, Tm: Annealing temperature			

### Ethical Considerations

The Ethics Committee of Shahid Sadoughi University of Medical Sciences, Yazd, Iran approved this experimental project (Code: IR.SSU.MEDICINE.REC.1400.316). The Declaration of Helsinki was followed when conducting the study. All individuals who took part in the study provided a written informed consent.

### Statistical Analysis

Data analysis was done by GraphPad Prism (version 8.4.3) and IBM SPSS Statistics (version 26, SPSS Inc., Chicago, USA). One-way ANOVA and multiple *t* tests were done to check the statistical differences between study groups. The significance was demonstrated by p < 0.05.

## 3. Results

### Demographic and clinical characteristics

40 women (20 in the DOR group and 20 in the control group) were included in this study. No statistically significant difference was observed between groups; however, the mean BMI of the DOR women was higher than control group (Table II).

### *CYP19A1*, *PKA*, *GSK3B* genes expression in CCs

**Figure 1:**
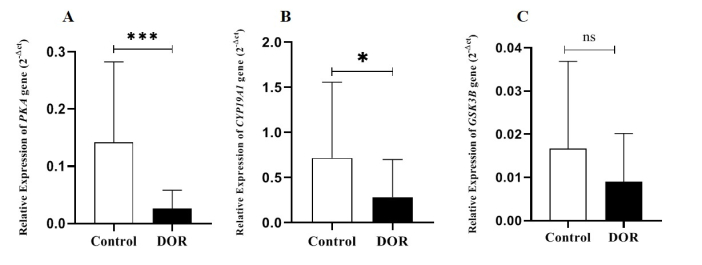
Expression of A) Protein kinase A (*PKA*) gene expression B) Cytochrome P450 aromatase (*CYP19A1*) gene expression, and C) Glycogen synthase kinase 3 beta (*GSK3B*) gene expression in the tested group compared with the control. DOR: Diminished ovarian reserve. *P < 0.05, ***P < 0.001, ns: Non-significant.

*CYP19A1* gene expression in DOR group was significantly lower than in the control group (p = 0.04). A significant difference was observed in the *PKA* gene expression in the DOR group compared to the control group (p < 0.001). Also, *GSK3B* gene expression was lower in the study group, although this difference was not statistically significant (p = 0.07). It was also demonstrated that the *PKA* and *GSK3B* gene expressions were lower in the study group, although these differences were not statistically significant. Our study's findings also demonstrated that the expression of the *PKA* gene had decreased in the study group (p = 0.0009) compared to control group. Also, expression of the *GSK3B* gene was lower in the study group, although this difference was not statistically significant (Figure 1).

**Figure 2:**
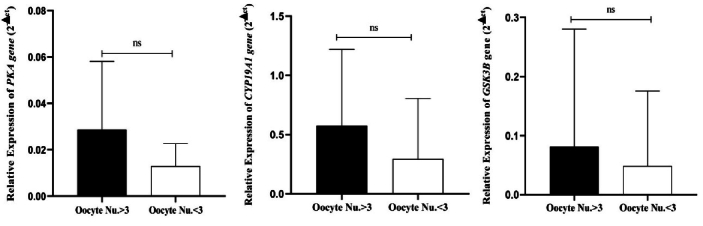
Expression of gene expression cytochrome P450 aromatase (*CYP19A1*),protein kinase A (*PKA*), andglycogen synthase kinase 3 beta (*GSK3B*)between DOR, with oocyte number < 3 and oocyte number > 3. ns: Non-significant.

It was also found that the expression of the *CYP19A1*, *PKA*, and *GSK3B* genes was lower in women with < 3 oocytes than in the other group. However, these differences were not statistically significant (Figure 2).

**Table 2:** Demographic information

**Variables**	**Control (n = 20)**	**DOR (n = 20)**	**P-value**
**Age (yr)**	31.88 ± 0.84	31.82 ± 0.80	0.689
**BMI (kg/m^2^)**	25.60 ± 3.43	27.01 ± 2.85	0.182
**FSH (mIU/mL)**	5.73 ± 2.50	9.22 ± 3.12	0.190
**AMH (ng/mL)**	3.75 ± 0.65	0.58 ± 0.08	< 0.001
**E2 (pg/mL)**	185 ± 13.72	203.02 ± 15.10	0.0232
**LH (mIU/mL)**	1.86 ± 0.35	3.51 ± 1.08	0.151
Data presented as Mean ± SD, *t* test. BMI: Body mass index, FSH: Follicle-stimulating hormone, AMH: Anti-Mullerian hormone, LH: Luteinizing hormone, E2: Estradiol, DOR: Diminished ovarian reserve			

## 4. Discussion

The results of our study show a decrease in the expression of the *CYP19A1* gene in a study group with poor ovarian response compared to the normal group. The essential steroidogenesis enzyme aromatase (P450 arom), which catalyzes the last stages of estrogen production from androgens, is encoded by the *CYP19A1* gene. In gonads and extragonadal tissue (brain and adipose tissue), it converts androstenedione to estrone and testosterone to estradiol and estrone (18). In granulosa cells, the aromatase enzyme is involved in the manufacture of estrogen. The quality of oocyte production, which in turn determines the quality of the embryo, is determined by the granulosa cells capacity to perform their roles. According to the study, women with polycystic ovary syndrome had lower average mRNA *CYP19A1* expression for aromatase in granulosa cells than women without polycystic ovary syndrome (19).

Many studies demonstrate that DOR in young women can be attributed to changes in the expression of specific genes, such as *CYP19A1* and *FSHR*, in cumulus and granulosa cells (20–22). *CYP19*-deficient mice have shown that this gene is crucial for the growth of ovarian follicles (23). Our findings also confirmed that the expression of the *PKA* gene had decreased in the DOR group as compared to the control group.

Numerous studies show the importance of the CAMP/PKA and Wnt signaling pathways in the production of estrogen, the growth of follicles, and ovulation (11–13). The expression of the *CYP19A1* gene rises as a result of *PKA*'s activation and effect on the beta-catenin signaling pathways of the Wnt signaling system. A study in 2016 found that ovulation is impacted by any nonfunctional *PKA* gene mutation (24).

Additionally, the Wnt signaling pathway is essential for the growth and division of follicles as well as ovulation, and its disruption affects both the process of ovulation and folliculogenesis (25). PKA/cAMP and PI3K-AKT signaling pathways have been linked to FSH-related signaling in GC (17, 26, 27). Our study's findings also demonstrated that the expression of the *GSK3B* gene decreased as compared to control group, although the expression difference was not statistically significant. In this article, we did not observe significant decreases in gene expression in women with fewer than 3 oocytes compared to women with more than 3 oocytes.

## 5. Conclusion

According to the findings, a defect in the expression of the *CYP19A1, GSK3B, *and* PKA* genes, as well as a disruption of the steroidogenesis process, are thought to be the causes of DOR and the variable responses of women to gonadotropin-releasing hormone inducing medications. Therefore, our data may offer new suggestions for molecular indicators to be used in studies of poor responders.

##  Data Availability

Upon a reasonable request, the corresponding author will provide the data supporting the study's conclusions.

##  Author Contributions

MH. Sheikhha and M. Lotfi designed the study and conducted the research. Z. Ahmadnia, F. Montazeri, and M. Lotfi monitored, evaluated, and analyzed the results of the study. Further, Z. Ahmadnia, S. Dashti, MH. Sheikhha, and M. Lotfi reviewed the article. All authors approved the final manuscript and take responsibility for the integrity of the data.

##  Conflict of Interest 

The authors declare that there is no conflict of interest.
